# Supplementary material for: Behavior change intervention to sustain iodide salt utilization in households in Ethiopia and study of the effect of iodine status on the growth of young children: community trial
Source: PeerJ. 2024 Mar 25;12:e16849. doi: 10.7717/peerj.16849 (PMC10977086; doi:10.7717/peerj.16849)
Supplement: Supplemental Information 3 [file peerj-12-16849-s003.docx]

We provided Consort Checklist for a trial as follow

Consort Checklist

| ***Subtopics*** | ***Item number*** |  | Pages |
| --- | --- | --- | --- |
| **Title and abstract** | 1a | Identification as a randomized trial in the title | Title Page |
|  | 1b | Structured summary of trial design, methods, results, And conclusions | Abstract |
| **Introduction** |  |  |  |
| Background and objectives | 2a | Scientific background and explanation of the rationale | 1-2 |
|  | 2b | Specific objectives or hypotheses | 2 |
| **Methods** |  |  |  |
| Trial design | 3a | Description of trial design (such as parallel, factorial) including allocation ratio | 3 |
|  | 3b | Important changes to methods after trial commencement (such as eligibility criteria), with reasons | 3 |
| Participants | 4a | Eligibility criteria for participants | 3 |
|  | 4b | Settings and locations where the data were collected | 3 |
| Interventions | 5 | The interventions for each group with sufficient details to allow replication, including how and when they were actually administered | 3 |
| Outcomes | 6a | Completely defined pre-specified primary and secondary outcome measures, including how and when they were assessed | 3 |
|  | 6b | Any changes to trial outcomes after the trial commenced, with reasons | 4 |
| Sample size | 7a | How sample size was determined? | 4 |
|  | 7b | When applicable, explanation of any interim analyses and stopping guidelines |  |
| Randomization |  |  |  |
| Sequence generation | 8a | The method used to generate the random allocation sequence | 4 |
|  | 8b | Type of randomization; details of any restriction (such as blocking and block size) | 4 |
| Allocation concealment mechanism | 9 | Teh mechanism used to implement the random allocation sequence (such as sequentially numbered containers), describing any steps taken to conceal the sequence until interventions were assigned | 4 |
| Implementation | 10 | Who generated the random allocation sequence, who enrolled participants, and who assigned participants to interventions | 4 -5 |
| Cluster masking /Blinding | 11a | If done, who was blinded after assignment to interventions (e.g., participants, care providers, those assessing outcomes) and how | 5 |
|  | 11b | If relevant, description of the similarity of interventions |  |
| measurements and Statistical | 12a | Statistical methods used to compare groups for primary and secondary outcomes | 5 |
| Statistical methods | 12b | Methods for additional analyses, such as subgroup analyses and adjusted analyses | 6 |
| **Results** |  |  | 6 |
| Participant flow (a diagram is strongly recommended) | 13a | For each group, the numbers of participants who were randomly assigned received intended treatment and were analyzed for the primary outcome | 6 |
|  | 13b | For each group, losses and exclusions after randomization, together with reasons | 6 |
| Recruitment | 14a | Dates defining the periods of recruitment and follow-up | 6 |
|  | 14b | Why the trial ended or was stopped | 6 |
| Baseline data | 15 | A table showing the baseline demographic and clinical characteristics for each group | Table 1 |
| Numbers analyzed | 16 | For each group, number of participants (denominator) included in each analysis and whether the analysis was by original assigned groups | 6 |
| Outcomes and estimation | 17a | For each primary and secondary outcome, results for each group, and teh estimated effect size and its precision (such as 95% confidence interval) | 6 |
|  | 17b | For binary outcomes, presentation of both absolute and relative effect sizes is recommended | Table 2 |
| Ancillary analyses | 18 | Results of any other analyses performed, including subgroup analyses and adjusted analyses, distinguishing pre-specified from exploratory | Table 3 |
| Harms | 19 | All important harms or unintended that effects in each group (for specific guidance see CONSORT for harms) |  |
| **Discussion** |  |  | 11 |
| Limitations | 20 | Trial limitations, addressing sources of potential bias, imprecision, and, if relevant, the multiplicity of analyses |  |
| Generalizability | 21 | Generalizability (external validity, applicability) of teh trial findings | 13 |
| Interpretation | 22 | Interpretation consistent wif results, balancing benefits and harms, and considering other relevant evidence | 14 |
| **Other information** |  |  |  |
| Registration | 23 | Registration number and name of trial registry | Abstract |
| Protocol | 24 | Where the full trial protocol can be accessed, if available | Title page |
| Funding | 25 | Sources of funding and other support (such as the supply of drugs), the role of funders | Title page |
